# Colestasis intrahepática por *Treponema pallidum* en paciente inmunocompetente

**DOI:** 10.7705/biomedica.6630

**Published:** 2023-06-30

**Authors:** Beatriz E. Orozco-Sebá, Diego Viasus, Esperanza Meléndez, Jairo Fuentes, José Tovar, Elkin A. Amado, Daniela Loaiza

**Affiliations:** 1 Programa de Dermatología, División de Ciencias de la Salud, Facultad de Medicina, Universidad del Norte, Barranquilla, Colombia Universidad del Norte División de Ciencias de la Salud Facultad de Medicina Universidad del Norte Barranquilla Colombia; 2 División de Ciencias de la Salud, Facultad de Medicina, Hospital Universidad del Norte de Barranquilla, Universidad del Norte, Barranquilla, Colombia Universidad del Norte División de Ciencias de la Salud Facultad de Medicina, Hospital Universidad del Norte de Barranquilla Universidad del Norte Barranquilla Colombia; 3 División de Ciencias de la Salud, Facultad de Medicina, Universidad del Norte, Barranquilla, Colombia Universidad del Norte División de Ciencias de la Salud Facultad de Medicina Universidad del Norte Barranquilla Colombia

**Keywords:** Treponema pallidum, sífilis, colestasis, terapéutica, Treponema pallidum, syphilis, cholestasis, therapeutics

## Abstract

La hepatitis por *Treponema pallidum* es una entidad poco frecuente y su diagnóstico representa un reto clínico. *Treponema pallidum* debe considerarse como etiología presuntiva en todo paciente con enfermedad hepática aguda, en el cual se hayan descartado otras causas más frecuentes.

Se presenta el caso de un paciente joven, inmunocompetente, quien presentó elevación de los valores de las pruebas hepáticas con patrón colestásico y lesiones maculopapulares en palmas y plantas. Dado su cuadro clínico, las pruebas diagnósticas y la respuesta a la terapia antimicrobiana instaurada, se estableció el diagnóstico de colestasis por una sífilis secundario sifilítiao. Es importante incluir la sífilis secundaria entre las posibles causas de enfermedad hepática aguda.

## Abstract (en)

Hepatitis due to *Treponema pallidum* is a rare entity and its diagnosis represents a clinical challenge. *Treponema pallidum* should be considered as a presumptive etiology in all patients with acute liver disease, when other frequent causes have been ruled out.

We present the case of a young, immunocompetent patient with elevated values in his liver tests, a cholestatic pattern, and maculopapular lesions on his palms and soles. Given his clinical picture, diagnostic tests, and response to the antimicrobial therapy, a diagnosis of cholestasis due to secondary syphilis has been established. It is important to include secondary syphilis within the possible causes of acute liver disease.

La sífilis es una enfermedad de transmisión sexual, causada por *Treponema pallidum*, la cual fue reconocida por primera vez en Europa durante el siglo XV ^1^. En la actualidad, los factores de riesgo para adquirir la enfermedad son la actividad sexual sin protección, la promiscuidad, la infección de transmisión sexual previa y el pertenecer a grupos vulnerables; los hombres representan el 86 % de los casos ^2^^,^^3^. En el 2020, en los Estados Unidos, se reportaron 133.954 casos de sífilis, con 41.655 de sífilis primaria y secundaria. Se observó un aumento del 6,8 % en comparación con el 2019 ^4^. Para el 2021, los datos preliminares arrojaron un aumento de los casos de sífilis primaria y secundaria en la población adulta, con un aumento del 34 % en mujeres y un incremento del 9 % en hombres ^5^.

Colombia dispone de un sistema de vigilancia de los casos de sífilis en mujeres gestantes y sus hijos, con una incidencia de sífilis congénita de 1,1 por 100 nacidos vivos. Sin embargo, hay pocos estudios que evalúen la sífilis en la población general o en los grupos de riesgo en Colombia ^6^. Los estudios realizados en bancos de sangre del país encontraron que la prevalencia de sífilis era del 0,93 % en Barranquilla y del 0,6 % en Medellín ^6^.

Aunque los estadios de la sífilis están bien definidos, las manifestaciones clínicas pueden variar mucho entre ellas, por lo que se le conoce como “la gran simuladora”. Por lo anterior, en algunos casos, la sífilis conlleva un reto diagnóstico ^2^^,^^7^. En la sífilis primaria se suele evidenciar una úlcera indolora que se resuelve de manera espontánea. En la sífilis secundaria se pueden encontrar lesiones papulares, eritematosas, presentes más frecuentemente en la región palmo-plantar. También encontramos alopecia en parches en el cuero cabelludo, de aspecto apolillado y lesiones tipo placas, en ocasiones eritematosas, en mucosa genital u oral ^4^. La sífilis terciaria aparece años después de la primera infección y para ese momento las afectaciones pueden ser neurológicas, cardíacas, óseas u oculares ^1^.

En relación con el diagnóstico de la enfermedad, las serologías se han convertido en el procedimiento más frecuente. Las pruebas no treponémicas miden simultáneamente inmunoglobulinas (Ig) G y M. Sin embargo, su positividad no asegura la enfermedad sifilítica. Por el contrario, las pruebas treponémicas producen escasos falsos positivos. Los ensayos de inmunoabsorción ligados a enzimas (ELISA) pueden emplearse en sustitución de las pruebas treponémicas de hemaglutinación (TPHA) y absorción de anticuerpos treponémicos fluorescentes (FTA-ABS) ya que han demostrado excelente sensibilidad y especificidad. Además, estas pruebas permiten la automatización de grandes cantidades de muestras y lecturas objetivas ^8^^,^^9^.

La hepatitis por *T. pallidum* usualmente se presenta con sintomatología inespecífica y suele ser asintomática. Su diagnóstico requiere evidencia bioquímica de lesión hepática en el contexto de serología treponémica confirmada y exclusión de otras posibles causas de hepatopatía ^7^^,^^10^. Los resultados de laboratorio suelen demostrar una alteración de los niveles de fosfatasa alcalina, transaminasas y bilirrubinas ^7^. Comúnmente se presenta hepatomegalia. Se espera un incremento acentuado de la fosfatasa alcalina y la gammaglutamil transferasa, con un modesto aumento de las transaminasas y bilirrubinas.

La biopsia hepática no es específica para hacer el diagnóstico. Sin embargo, se puede observar un infiltrado linfocítico periportal con necrosis focal de células hepáticas. La presencia de espiroquetas en la biopsia hepática es diagnóstica, pero es poco frecuente encontrarlas ^11^.

Se presenta el caso de un hombre adulto que consultó por un cuadro clínico inicial de dolor abdominal acompañado de ictericia, coluria y acolia; además, presentaba lesiones eritematosas maculopapulares en palmas y plantas. Los resultados de laboratorio evidenciaron alteraciones de la función hepática con patrón colestásico.

Dado su cuadro clínico, los resultados de las pruebas diagnósticas y la respuesta a la terapia antimicrobiana instaurada, se estableció el diagnóstico de colestasis por una sífilis secundaria.

## Presentación del caso

Se trata de un paciente masculino de 31 años, que acudió al servicio de urgencias en marzo de 2022. Presentaba un cuadro clínico de seis días de evolución de dolor abdominal tipo urente, asociado a astenia, adinamia, coluria, acolia y prurito generalizado. Durante la anamnesis, el paciente refirió haber tenido relaciones sexuales con hombres, negó antecedentes de enfermedades de transmisión sexual y manifestó haber tenido un solo compañero sexual en los últimos seis meses. El paciente negó la presencia de alguna lesión primaria mucocutánea en genitales, ano o boca. Como antecedentes médicos tiene hipertrigliceridemia familiar y trastorno depresivo desde hace dos años, manejado con antidepresivos.

**Figura 1:**
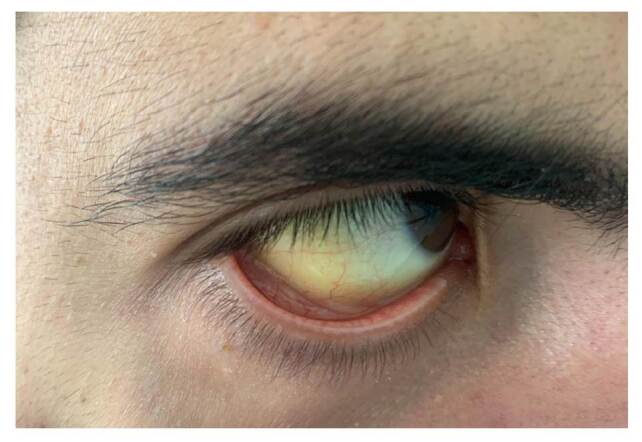
Escleróticas ictéricas

**Figura 2:**
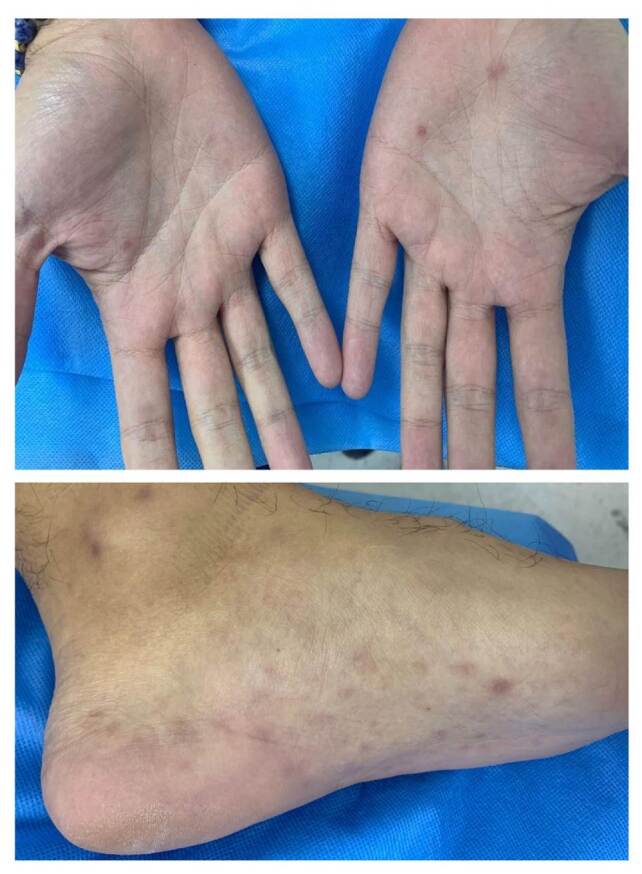
Exantema maculopapular en palmas y plantas

 En el examen físico se observó un tinte amarillento en los ojos, escleróticas ictéricas (figura 1), y costras en la piel por prurito generalizado. Además, se evidenciaron lesiones maculopapulares en palmas y plantas (figura 2). En los resultados de laboratorio se documentó la elevación de las transaminasas en un valor no mayor de diez veces el valor superior normal, con predominio de la alanina-aminotransferasa sobre la aspartato- aminotransferasa. También se encontró aumento de la fosfatasa alcalina e hiperbilirrubinemia a expensas de la conjugada. En el cuadro 1 se muestran los resultados de las pruebas de laboratorio.

**Cuadro 1:** Perfil bioquímico hepático del paciente, antes y después del inicio del tratamiento

Prueba de laboratorio	24/03/2022	30/03/2022	31/03/2022*	07/04/2022	14/04/2022
Transaminasa glutámico oxaloacética (U/L)	96,8	111,21	133,06	97,55	101,90
Transaminasa glutámico-pirúvica (U/L)	265,6	225,81	246,78	202,21	189,84
Fosfatasa alcalina (U/L)	1814,2	1684,97	-	1256,43	859,69
Gamma-glutamil transferasa (U/L)	-	305,53	-	-	-
Bilirrubina total (mg/dl)	3,0	5,13	5,62	2,89	1,86
Bilirrubina directa (mg/dl)	3,17	4,43	4,96	2,28	1,19
Tiempo de protrombina (s)	9,1	-	9,8	11,5	8,6
Tiempo parcial de tromboplastina (s)	48,0	-	52,1	35,8	31,9
RPR (diluciones)	-	1/128	-	-	-
FTA-ABS	-	Positivo	-	-	-
Anticuerpos para HIV	-	Negativo	-	-	-

- no aplica, no se practicó en esta fecha

* Inicio de penicilina G benzatínica a dosis de 2,4 x 10^6^ UI

Ante los hallazgos anteriores, se ordenó una ecografía abdominal total que reportó hepatomegalia con dilatación de la vía biliar intrahepática, y una pancreatocolangiografía por resonancia magnética en la que no se evidenciaron anormalidades (cuadro 2).

**Cuadro 2:** Resultados de las imágenes diagnósticas

Imagen diagnóstica	Resultados
Ecografía de abdomen total	Hepatomegalia con dilatación de la vía biliar intrahepática. Se observa aumento de tamaño del hígado con longitud craneocaudal de 18,8 cm de contornos y ecogenicidad normal. Las vías biliares intrahepáticas muestran leve dilatación hacia el lado izquierdo del hígado.
Pancreatocolangiografía por resonancia magnética	Normal. Se descarta compromiso intrahepático y extrahepático.

Asimismo, se solicitaron serologías en búsqueda de etiología de enfermedad hepática aguda (IgM para herpes I y II, hepatitis A, *Toxoplasma gondii* y Epstein Barr; anticuerpos contra la nucleocápside del virus de la hepatitis B, antígeno de superficie para la hepatitis B, anticuerpos contra VIH y hepatitis C). Todos los reportes de las serologías fueron negativos.

A los seis días de la asistencia del paciente al servicio de urgencias, se practicaron nuevas pruebas de laboratorio de manera ambulatoria. Se encontró persistencia de la alteración del perfil hepático (cuadro 1), con una mayor hiperbilirrubinemia a expensas de la directa.

En la revisión del paciente en el Servicio de Dermatología se evidenciaron lesiones eritematosas maculopapulares en la región palmar y en la plantar de, aproximadamente, 0,5 x 0,2 cm (figura 2). La reagina plasmática rápida (RPR) presentó reactividad en la dilución 1/128, la FTAABS con resultado positivo y la prueba de VIH de cuarta generación con resultado negativo. También se reportó IgM positiva para citomegalovirus.

El paciente fue tratado con tres dosis de penicilina G benzatínica intramuscular de 2,4 x 10^6^ UI, administradas semanalmente. Luego del inicio de la terapia antibiótica se observó mejoría clínica y descenso de los valores de las pruebas hepáticas (cuadro 1).

### Consideraciones éticas

El paciente autorizó el uso de los datos clínicos y de las imágenes mediante un consentimiento informado.

## Discusión

El caso evidencia un paciente con ictericia, coluria, acolia y lesiones maculopapulares en palmas y plantas. En los exámenes de laboratorio se documentó un patrón colestásico sin evidencia de obstrucción de la vía biliar en imágenes diagnósticas. Los resultados de las pruebas etiológicas fueron positivos para *T. pallidum*. El paciente tuvo una rápida mejoría en los hallazgos clínicos y las alteraciones de laboratorio después del tratamiento con penicilina G benzatínica.

Actualmente ha aumentado la incidencia de enfermedades de transmisión sexual a nivel mundial lo cual ha generado un problema de salud pública. Estas enfermedades suponen un reto diagnóstico, siendo importante identificarlas de manera temprana para formular un tratamiento oportuno ^1^^,^^12^^,^^13^. Además, es esencial realizar intervenciones y profilaxis no farmacológicas en los pacientes con factores de riesgo para disminuir la morbilidad y mortalidad asociadas a las enfermedades de transmisión sexual. Se debe brindar educación sexual y asesoría sobre prácticas más seguras como el uso de preservativos. Además, se recomienda la vacunación contra el virus de las hepatitis A, B y C, y el virus del papiloma humano ^8^^,^^14^.

Aunque la hepatitis por *T. pallidum* es una entidad poco frecuente, la sífilis tiene la capacidad de comprometer cualquier órgano o sistema ^14^. El primer caso de compromiso hepático por sífilis secundaria se describió en 1918 ^11^. Se han reportado pocos casos en las últimas décadas de compromiso hepático por sífilis, siendo más frecuente la hepatitis y menos frecuente la colestasis ^15^^,^^16^.

Se han considerado varios criterios diagnósticos de hepatopatía por sífilis secundaria en diversos artículos, como son las pruebas no treponémicas y treponémicas positivas, el perfil hepático alterado con aumento de la fosfatasa alcalina, las transaminasas y las bilirrubinas, la ausencia de otras causas alternativas más frecuentes y la completa recuperación clínica tras el inicio del tratamiento con penicilina ^3^^,^^12^^,^^14^.

Se ha sugerido que en la sífilis secundaria ocurre diseminación hasta el hígado por vía hematógena que va desde el sistema venoso hasta la circulación portal ^13^^,^^14^. La hepatopatía por *T. pallidum* se puede presentar en la sífilis secundaria y terciaria ^12^. Cuando ocurre en el último estadio de la sífilis, puede manifestarse como cirrosis, insuficiencia hepática o hepatitis fulminante ^1^. Aún no se conoce claramente la patogénesis del daño hepático. Sin embargo, se sugieren dos teorías: un mecanismo de autoinmunidad hepática basado en la presencia de anticuerpos contra los hepatocitos infectados, o una invasión hepática por las espiroquetas en la fase de diseminación que generan daño canalicular ^1^^,^^12^^,^^16^.

En este caso, no hubo caracterización histopatológica del daño hepático. No obstante, el diagnóstico histológico de colestasis por *T. pallidum* es poco específico y el hallazgo de las espiroquetas en el tejido hepático es menor del 50 % ^16^.

## Conclusión

La colestasis por *T. pallidum* es de difícil diagnóstico por su baja frecuencia. La colestasis por *T. pallidum* suele presentarse con signos y síntomas inespecíficos. Por esto es clave sospechar esta etiología cuando se descartan las causas más frecuentes de hepatopatía colestásica estructural u otros tipos de enfermedades infecciosas diferentes a la sífilis.

Con este caso observamos la importancia del examen físico en la historia clínica y su correlación con las serologías y respuesta al tratamiento para confirmar el diagnóstico. Es importante incluir la sífilis secundaria entre las posibles causas de enfermedad hepática aguda.
