# Enmienda

**Published:** 2024-05-30

En relación con el artículo “Colestasis intrahepática por *Treponema pallidum* en paciente inmunocompetente” publicado en Biomédica. 2023:43(4):164-70, en la sección de discusión (p. 168, párrafo 2, renglones 9 y 10) se identificó un error que requiere corrección.

Dice: “[...] Además, se recomienda la vacunación contra el virus de las hepatitis A, B y C, y el virus del papiloma humano (8,14) [...]”.

Debe decir: “[…] Además, se recomienda la vacunación contra el virus de las hepatitis A y B y la del virus del papiloma humano, según las indicaciones de las guías locales o internacionales (8,14). Pese a que se han realizado estudios con algunos candidatos potenciales, a la fecha no se dispone de ninguna vacuna para la hepatitis C […]”.

Los autores agradecen la oportunidad de corregir el error, nos disculpamos por cualquier confusión que este error pudo haber causado y aprecian la comprensión de los lectores.

Un cordial saludo,

Beatriz E. Orozco-Sebá

Diego Viasus

Esperanza Meléndez

Jairo Fuentes

José Tovar

Elkin A. Amado

Daniela Loaiza

